# A Sparse-Driven Anti-Velocity Deception Jamming Strategy Based on Pulse-Doppler Radar with Random Pulse Initial Phases

**DOI:** 10.3390/s18041249

**Published:** 2018-04-18

**Authors:** Zhen Liu, Jinping Sui, Zhenhua Wei, Xiang Li

**Affiliations:** 1 College of Electronic Science, National University of Defense Technology, Changsha 410073, China; zhen_liu@nudt.edu.cn (Z.L.); lixiang01@vip.sina.com (X.L.); 2 Rocket Force University of Engineering, Xi’an 710025, China; wzh016001@aliyun.com

**Keywords:** anti-velocity false target jamming, random pulse repetition interval, pulse diversity, electronic counter-countermeasures

## Abstract

This paper focuses on developing an anti-velocity jamming strategy that enhances the ability of a pulse-Doppler (PD) radar to detect moving targets in the presence of translational and/or micro motion velocity jamming generated by the digital radio frequency memory (DRFM) repeat jammers. The strategy adopts random pulse initial phase (RPIP) pulses as its transmitted signal and thus gets DRFM jammers not adaptable to the randomness of initial phase of the transmitted pulses in the pulse repetition interval (PRI) domain. The difference between the true target echo and the false target jamming signal at each PRI is then utilized to recognize the true and false target signals. In particular, an entropy based multi-channel processing scheme is designed to extract the information of the received signal without the assumption that true and false targets must be both included within one coherent processing interval (CPI). Information such as the component of the received signal (target echo only, jamming only or both) or the operating manner of DRFM repeat jammer can be gained (if jamming exists). Meanwhile, we solve the false target recognition problem under sparse theory frame and our previous work named the short-time sparse recovery (STSR) algorithm is introduced to recover the motion parameters of the true and/or false targets in the time-frequency domain. It should be pointed out that both the translational false target jamming and micro motion target jamming can be recognized in our strategy. The performance of the proposed strategy is compared with the correlated processing (CP) method used by most extant strategies. It is shown that the proposed strategy can successfully recognize the existence of true and/or false targets and keep its power in recovering corresponding motion parameters even when the jamming environment is strong.

## 1. Introduction

Pulse-Doppler (PD) radars are radar systems that utilize moving target detection (MTD) or moving target indication (MTI) techniques to detect targets in the midst of noise, clutter and jamming [1,2,3]. Essentially, these systems are based on the fact that moving targets induce frequency modulations on the detecting signal, known as Doppler effect or Doppler frequency shift [3,4,5,6,7]. By extracting the Doppler frequency shift, radars can measure the radial velocity of the moving object. With the remarkable capability of distinguishing between slowly moving or relatively stationary targets and fast moving ones [1], PD radars are widely used in civilian and military fields, such as air surveillance, ground moving target recognition and the detection of low aircraft [3,8,9].

Meanwhile, the prominent usage of these radars in the military has directly contributed to the rapid development of the corresponding jamming techniques, namely velocity deception jamming (VDJ), in electronic countermeasures (ECM) fields. Especially with the development and maturity of digital radio frequency memory (DRFM) techniques, the DRFM-based jammer, also called DRFM repeat jammer [3,10,11,12], is capable of generating deceptive/false velocity targets by interrupting, storing, modulating and repeating the radar’s transmitted signal. In addition to jamming the detection of radar on translational motion targets by generating translational false targets (TFT), recent research [13,14,15] has shown that modern VDJ techniques have accomplished the generation of the micro motion false targets (MMFT) by modulating additional micro-Doppler frequency shift besides the Doppler frequency shift on its jamming signals. Hence, DRFM-based VDJ techniques severely threaten the survival of the PD radars in modern warfare.

Countering DRFM-based velocity deception jamming has been one of the hottest topics in the electronic counter-countermeasures (ECCM) field. Researchers solve the problem by proposing different strategies based on pulse diversity [3,10,16,17], polarization [18], DRFM quantization [19,20,21], etc. Pulse diversity is commonly considered to be the most effective method to counter the DRFM-based jamming [3,10]. Pulse diversity refers to the technique that the pulses transmitted by the radar vary at each pulse repetition interval (PRI) and such variation is only known to the radar [3,10]. The DRFM repeat jammer needs to analyze the intercepted pulse before retransmitting so it chooses to utilize the pulse intercepted at previous PRI or some other past PRI to generate the jamming signal at current PRI. Therefore, the pulse retransmitted by DRFM differs from the pulse transmitted by the radar at a given PRI. Aiming at enlarging and/or utilizing the difference, various pulse-diversifying methods are currently being researched to counter DRFM-based false velocity jamming [3,17,22,23,24]. Refs. [3,22,23] all utilized the adaptive initial phases pulses as the transmitted signals and recognized the true target(s) from the false targets via the obvious differences of two kinds of targets in the frequency domain. Ref. [10] proposed a pulse diversity scheme that varies the rate of the chirp or the phase at each PRI and suppressed the DRFM repeat jamming in the range-Doppler domain. Though numerous pulse diversifying measures have been proposed to suppress the jamming signal in order to detect the target and estimate the targets’ parameters further, this subject is far from well-researched. In particular, firstly, most of the extant methods are based on the assumption that the jamming signal and target echo must exist simultaneously in one coherent processing interval (CPI) while ignoring the cases that only jamming signal or true target echo is received in reality. Here, we just take velocity gate pull off (VGPO) jamming as an example [25]. Only a true target exists at the first stage (when the jammer just retransmits the signal of the true targets) or the last stage (when the jammer is shut off) of the jamming. Secondly, only the TFT jamming is considered in most approaches that cannot directly be used to suppress the MMFT jamming. Thirdly, most researches adopt the traditional detection methods [3,10], i.e., correlated processing (CP), to process the pulse-diversifying signal (a random or a quasi-random signal), which will lead to a higher side lobe.

Actually, we have successfully proposed a sparse-based method to counter TFT [26,27] and MMFT [28] in our previous work. The basic idea can be summarized as follows. Under the sparse representation theory, we built two corresponding dictionaries (called true target and false target sparse representation dictionary respectively) and meanwhile the true target echo or false target jamming signal can only be represented sparsely under their own dictionary. With the help of sparse theory, the two kinds of signals can be separated. Meanwhile, we have already proved that the sparse recovery performs better when processing the random signal than CP-based methods. However, though promising results have been gained, the method needs to be optimized to be capable of automatically recognizing the components of the received signal and countering both TFT and MMFT jamming.

In this paper, a more powerful sparse-driven anti-velocity deception jamming strategy is proposed based on our previous work. The random pulse initial phase (RPIP) signal is adopted as the transmitted signal by the radar. The proposed strategy can counter both TFT and MMFT jamming generated by the DRFM repeat jammer. Moreover, the components of the received signal will be automatically recognized and the motion parameters of true target and/or false target will be recovered in the time-frequency domain. Firstly, an entropy-based multi-channel processing scheme is designed to analyze the received signal about its components. Meanwhile, the information about the DRFM repeat jammer operating manner will be extracted if the jamming exists. Then, the corresponding dictionaries for the true and false targets will be built based on the information pre-known (for true target) and/or extracted (for false target). At last, the motion parameters will be recovered in the time-frequency domain by a short-time sparse representation algorithm (STSR), which was proposed in our previous work [29].

The paper is organized as follows. In Section 2, the problem that moving target detection using an RPIP signal is formulated and the target echo and the jamming signal are modeled. In Section 3, we propose an entropy-based multi-processing scheme to extract the information from the received signal. A method is also propose here to build the sparse representation dictionaries for the true target echo and jamming signal. Meanwhile, the STSR algorithm is introduced to get the two signals separated. Section 4 carries out some comparative simulations to testify to the performance and superiority of our proposed strategy and the study concludes in Section 5.

## 2. Problem Formulation

### 2.1. Transmitted Signal and Target Echo

**Figure 1:**
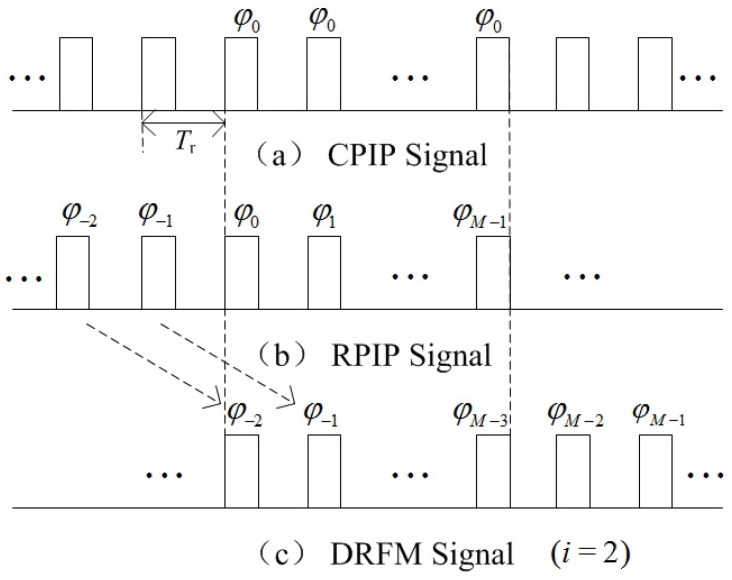
The comparison of three kinds of signals with different phase modulations. (**a**) CPIP (Constant Pulse Initial Phase) signal; (**b**) RPIP signal; (**c**) DRFM repeat jamming signal (i=2).

Different from pulses with constant initial phase (CPIP), RPIP signals belong to random modulated signals, whose phases vary at each PRI. Figure 1a,b illustrate differences between CPIP signal and RPIP signal during a CPI briefly.

Now, we assume that the radar system transmits a RPIP signal, as is shown in Figure 1b. The transmitted signal during a CPI can be represented as

(1) $$s^{t}\left(t\right)=\frac{1}{\sqrt{MT}}\sum_{m=0}^{M-1}rect\left(\frac{t-mT_{r}}{T}\right)exp\left(j2\pi f_{0}t+j\varphi_{m}\right),$$

where *M*, *T*, $T_{r}$ and $f_{0}$ denote the number of pulses in a CPI, the pulse width, PRI and carrier frequency, respectively. Function rect(•) is the rectangular envelope of the transmitted pulse and $\varphi_{m}$ is the initial phase of the *m*th pulse, which is assumed to follow a certain distribution and statistically independent of that of other pulses.

Assume that there is a moving target (denoted as $O^{r}$ ) in the radial direction of radar. The initial distance between target $O^{r}$ and radar is denoted as $R_{0}^{r}$. Note that not only the translational motion but also micro motion are considered in this paper. Thus, the motion of $O^{r}$ can be divided into two parts: one is translational motion, i.e., $O^{r}$ has relative motion for radar at a radial velocity $v^{r}\left(t\right)$. It should be pointed out that $v^{r}\left(t\right)$ will be positive when $O^{r}$ is approaching the radar and be negative when $O^{r}$ is away from the radar. The other one is the micro motion. Assume that there are *P* scattering centers in total and they are all making micro motion in addition to the bulk motion of $O^{r}$. It should be noted that, in this paper, we mainly consider the case that the *P* scattering centers are moving in the same range gate. Then, the echo returned from $O^{r}$ and received by the radar at the *m*th PRI can be expressed as Equation (2) after being mixed with $s^{t*}\left(t\right)$

(2) $$s_{m}^{r}\left(t\right)=\sum_{p=1}^{P}\frac{A_{p}^{r}}{\sqrt{MT}}rect\left(\frac{t-mT_{r}-t_{p}^{r}}{T}\right)exp\left(-j2\pi f_{0}t_{p}^{r}\right)+e^{r}\left(t\right),$$

where $A_{p}^{r}$, $t_{p}^{r}$ and $e^{r}\left(t\right)$ denote the reflectivity coefficient, delay time of the *p*th scattering center and observation noise, respectively. Considering the existence of the jamming signal, we use $\varphi_{m}^{r}$ to denote the initial phase of the *m*th of received pulse from target for avoiding confusion. It also should be pointed out that $\varphi_{m}^{r}$ is cancelled during the mixing process. Additionally, $t_{p}^{r}$ can be expressed as

(3) $$t_{p}^{r}=2\left(R_{0}^{r}+R^{r}\left(t\right)+R_{p}^{r}\left(t\right)\right)/c,$$

where $R^{r}\left(t\right)$ denotes the instantaneous distance from the radar receiver to $O^{r}$ and $R_{p}^{r}\left(t\right)$ denotes the instantaneous distance change that arises from the scattering centers. Note that, if no micro motion exists, $R_{p}^{r}\left(t\right)$ will be equal to 0, i.e., $O^{r}$ just has translational motion.

### 2.2. Velocity False Target Jamming Signal

In this paper, the velocity false target refers to the jamming signal emitted by the DRFM repeat jammer that is set on the moving target (called self-defense jammer) to protect the target itself. To this end, the jammer needs to conduct the following steps to generate a jamming signal that can be coherent to the transmitted signal of the radar and form the false velocity targets after being processed. Firstly, the transmitted pulse $s^{t}$ is intercepted. Then, the key parameters such as carrier frequency, pulse width, initial phase, etc., are obtained. Thirdly, translational modulation and micro motion modulation function are generated based on the information gained, and the intercepted pulse is modulated by the functions. Finally, the modulated pulse will be retransmitted [13]. Based on this work flow, the jamming pulses are coherent to the pulses transmitted by radar and easy to get the radar processing gain. However, when the radar transmits RPIP signal, the jammer cannot adapt easily to the change of the pulse initial phase in each PRI. Hence, the pulse emitted by the jammer will lag *i*PRI (i=0,1,…,M−1) behind the pulse transmitted by the radar [3]. Concretely, at the *m*th PRI, the jammer emits the modulated pulse, which is intercepted in the (m−i)th PRI. As for the case that m<i, the emitted pulses will be generated based on the corresponding pulses intercepted during previous CPI, or generated randomly (at the beginning period of jamming). Figure 1b,c show the RPIP pulses transmitted by the radar and the typical case of the jamming pulses received in one CPI (when i=2).

For the receiver of radar, the DRFM repeat jammer generates false velocity targets (denoted as $O^{d}$) via the method mentioned above. The received jamming pulse at the *m*th PRI of radar can be expressed as Equation (4) after being mixed

(4) $$s_{m}^{d}\left(t\right)=\sum_{q=1}^{Q}\frac{A_{q}^{d}}{\sqrt{MT}}rect\left(\frac{t-mT_{r}-t_{q}^{d}}{T}\right)exp\left(-j2\pi f_{0}t_{q}^{d}+j\left(\varphi_{m}^{d}-\varphi_{m}^{r}\right)\right)+e^{d}\left(t\right),$$

where *Q*, $A_{q}^{d}$, $t_{q}^{d}$ denote the number, the reflectivity coefficient, and the delay time of the scattering centers of the jamming target $O^{d}$, respectively. $\varphi_{m}^{d}$ denotes the initial phase of the *m*th jamming pulse. As already mentioned, the initial phases of pulses back from the jammer $\varphi^{d}$ in one CPI can be viewed as a lag *i*PRI behind those of the pulses from the (true) target, i.e., $\varphi^{r}$. We formulate this lag as a function named Lag(•), which is $Lag(\varphi^{r},\varphi^{d})=i$. The parameter *i* is named the lag information in this paper and it should be pointed that *i* is of paramount importance for recognizing the jamming pulses, which will be explained further later. The $t_{q}^{d}$ stands for the modulation part from the jammer. Generally, it can be expressed as Equation (5)

(5) $$t_{q}^{d}=2\left(R_{0}^{d}+R^{d}\left(t\right)+R_{q}^{d}\left(t\right)\right)/c,$$

where $R_{0}^{d}$ is the initial distance set by the jammer. $R^{d}\left(t\right)$ and $R_{q}^{d}\left(t\right)$ are directly decided by the translational and micro motion of the false target and its scattering center, respectively.

## 3. An ECCM Strategy Based on Sparse Representation Theory

We have formulated the two kinds of signals that the radar will receive under the velocity jamming scenarios. It is difficult to distinguish the target echo (denoted as $s^{r}$) and the jamming signal (denoted as $s^{d}$) because the DRFM jamming signals can easily get into the receiver and get the processing gain after the matching filter. According to the sparse theory, if we can build two dictionaries $U^{r}$ and $U^{d}$, and $s^{r}$ ($s^{d}$) can be only be sparsely represented under $U^{r}$ (correspondingly $U^{d}$), then we can get $s^{r}$ and $s^{d}$ be separated. It is noticeable that the difference of the pulse initial phases have the potential to help us to distinguish the two signals when comparing Equations (2) and (4). In this section, we propose a velocity false target recognition strategy that can separate the true target echo and the jamming signal.

### 3.1. Information Extraction Based on Entropy by Multi-Channel Processing

**Figure 2:**
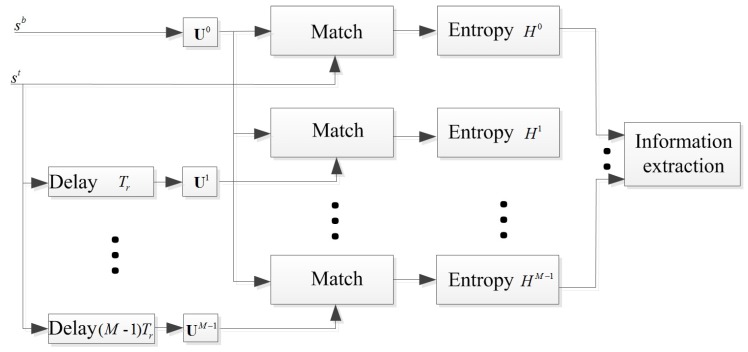
Entropy-based multi-channel processing scheme for analyzing the received signal.

For most extant antivelocity jamming methods, fundamental assumptions are that the existence of the jamming is pre-known or the jamming and the true target echoes are always being received in the same CPI. Obviously, these are not always the cases in real world scenarios. The first requirement for an ECCM scheme should be judging the component of the receiving signal (denoted as $s^{b}$), i.e., (i) only the target echo ($s^{r}$); (ii) only the jamming signal ($s^{d}$); (iii) or that both $s^{r}$ and $s^{d}$ exist simultaneously. This would be more realistic for an ECCM when working on a battle field. Meanwhile, if the jamming exists, the lag information should be extracted for further processing. Hence, a multi-channel matched filtering pre-processing mechanism is designed for extracting the basic information about the jamming. The corresponding mechanism of the multi-channel preprocessing is plotted in Figure 2.

Considering the mentioned operating manner of the repeat-back jammer, i.e., the jamming pulses $s^{d}$ are generated based on the transmitted pulses $s^{t}$ with a certain lag PRI (for example $i_{0}$), $s^{d}$ must be matched well with the pulses with lag $i_{0}$PRI compared other pulses ($i\neq i_{0}$). Consequently, as shown in Figure 2, all the possible *i* values are taken into consideration. The $s^{b}$ is matched with a specific matrix $U^{l}$ in the *l*th (l=0,1,…,M−1) channel. Note that, in this paper, we mainly consider the case that the range and velocity are both unambiguous. The unambiguous velocity is $v_{u}=\lambda /2T_{r}$ (λ is the wavelength of the radar signal). We divide $v_{u}$ into *G* parts uniformly and then the velocity resolution is $\Delta v=v_{u}/G$. For the *l*th channel, the corresponding matrix $U^{l}$ is built of which elements are $U_{mg}^{l}=exp\left(j4\pi f_{0}mT_{r}g\Delta v/c+j\varphi_{m-l}\right)$ (g=0,1,…,G−1). Then, when we input $s^{b}$ into the *l*th channel, the corresponding matching result $s_{H}^{l}$ can be expressed as Equation (6):

(6) $$s_{H}^{l}=s^{b}U^{l}={\left[s_{0}^{l},s_{1}^{l},\cdots ,s_{g}^{l},\cdots ,s_{G-1}^{l}\right]}_{1\times G}.$$

Then, the Shannon information entropy $H^{l}$ of $s_{H}^{l}$ is calculated by Equation (7):

(7) $$H^{l}=-\sum_{g=0}^{card(s_{H}^{l})-1}P\left(s_{g}^{l}\right)log_{2}P\left(s_{g}^{l}\right),$$

where P(•) is the possibility function, and $card(s_{H}^{l})$ denotes the number of different elements in $s_{H}^{l}$. It should be pointed that $P(s_{g}^{l})$ corresponds to the frequency that $s_{g}^{l}$ occurs in $s_{H}^{l}$. The frequency $f^{e}$ of $s_{g}^{l}$ is calculated by Equation (8)

(8) $$P\left(s_{g}^{l}\right)=f^{e}\left(s_{g}^{l}\right)=\frac{z_{g}^{l}}{G},$$

where $z_{g}^{l}$ denotes the number of occurrence of $s_{g}^{l}$ among $s_{H}^{l}$. Here information entropy is used to quantitatively represent the randomness of a matching result $s_{H}^{l}$. By this approach, we can gain an entropy set $H=\{H^{0},H^{1},\cdots ,H^{l},\cdots ,H^{M-1}\}$ when we successively put $s^{b}$ into each channel. We call the H the entropy spectrum. According to the property of the entropy, a smaller entropy indicates that $s^{b}$ is more correlated with the $U^{l}$.

**Figure 3:**
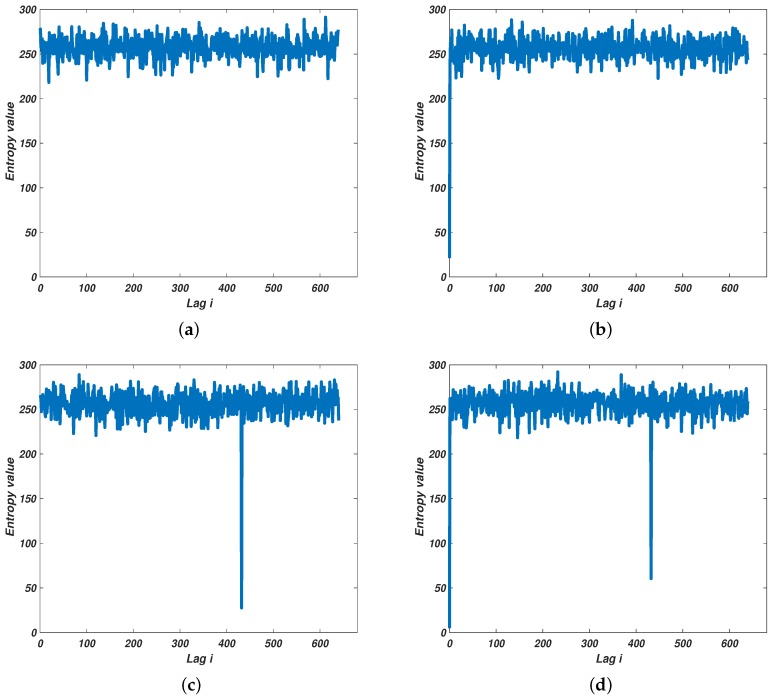
The outputs of multi-channel processing scheme (M=640). (**a**) No echo detected; (**b**) Only $s^{r}$ exists (i=423); (**c**) Only $s^{d}$ exists (i=0); (**d**) $s^{r}$ and $s^{d}$ exists simultaneously (i=0 and 423).

Now let us reconsider the possible cases for the component of $s^{b}$. When $s^{b}=s^{r}$, we have the lowest value in the entropy spectrum i.e., $H^{0}$ is the lowest value in H. If $s^{b}=s^{d}$ and $Lag\left(\varphi^{r},\varphi^{d}\right)=i_{0}$, the corresponding entropy $H^{i_{0}}$ will be the lowest value. As for the case that $s^{b}=s^{r}+s^{d}$, there will be two negative peaks in the entropy spectrum. Besides the component of $s^{b}$ being detected, the lag information will also be extracted by this multi-channel approach. Typical entropy based information extraction approach results are illustrated in Figure 3.

Figure 3a–d illustrate the cases that the component of $s^{d}$ could be. The *x*-axis and the *y*-axis of Figure 3a–d correspond to possible lag information, i.e., *i*, and the corresponding entropy value for each possible *i*. An additional case is shown for better comparison that no signal is received (see Figure 3a). Accordingly, if no output from a certain channel is much lower that the rest (see Figure 3a), it indicates that no target or jamming is detected. If the output of the first channel is the only negative peak (one which is considerably lower than the rest) in the entropy spectrum, then only $s^{r}$ exists. (corresponding to Figure 3b). When the lowest output is from other channel but not the first channel and the first channel’s output is not much lower than the others (as shown in Figure 3c), only $s^{d}$ exists. As for the last case, two outputs (one is from the first channel) are the two much lower points in the entropy spectrum, which denotes that $s^{r}$ and $s^{d}$ exist simultaneously.

### 3.2. Motion Parameters Separation and Recovery Based on Sparse Representation Theory

**Figure 4:**
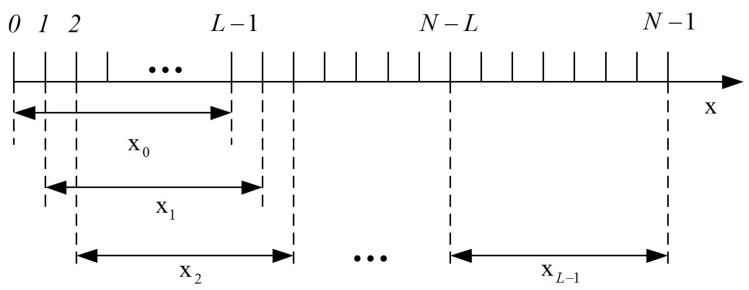
The main idea of the STSR (Short-time Sparse Representation) algorithm.

The micro-Doppler effect has time-varying and periodic properties [6,7,30], so the Fourier transform is not suitable for extracting time-dependent information of target and jamming signals. In this paper, we employ a time-frequency method named STSR that was proposed in our previous work [29]. Here, the main idea is given (depicted in Figure 4). Interested readers can refer to [29] for more details about STSR.

Assume x=[x[0],x[1],⋯,x[N−1]] is a discrete signal. There is a rectangular sliding window function w(n)=u(n)−u(n+L−1) of which the length is *L*. u(k) is the unit step function. Substantially, time-frequency analysis (TFA) is actually to find a representation in Fourier domain for the windowed signal. The main idea of the STSR algorithm is getting the sparse representation of discrete signal x in the frequency domain at each time instant and then synthesising the results [29]. At each time instant k(k=0,1,⋯,L−1), we have

(9) $$x_{k}=x*w\left(k\right).$$

Assume that the Φ is a dictionary under which the $x_{k}$ can be represented sparsely. According the sparse theory, we then have $x_{k}={\left(\Phi y_{k}\right)}^{T}$. $y_{k}$ is called the sparse representation of $x_{k}$ under Φ. $y_{k}$ can be obtained by working out the convex optimization problem as follows:

(10) $$\hat{y_{k}}=\arg\min\;∥\;y_{k}\;{∥}_{0}s.t.∥\;{\left(\Phi y_{k}\right)}^{T}-x_{k}\;{∥}_{2}\leq \varepsilon ,$$

where $∥\;\;{∥}_{0}$ and $∥\;\;{∥}_{2}$ denote the $l_{0}$-norm (i.e., the number of nonzero components in the vector) and $l_{2}$-norm, $\hat{y_{k}}$ indicates the estimated frequency distribution of the signals, and ε is the fitting error threshold. Obviously, direct optimization of Equation (10) is an NP-hard (non-deterministic polynomial-time hard) problem. We therefore do a convex relaxation of the problem by using $l_{0}$-norm to replace $l_{1}$-norm. Thus, the optimization problem of Equation (10) is relaxed into the following optimization problem:

(11) $$\hat{y_{k}}=\arg\min\;∥\;y_{k}\;{∥}_{1}s.t.∥\;{\left(\Phi y_{k}\right)}^{T}-x_{k}\;{∥}_{2}\leq \varepsilon .$$

There have been various kinds of methods proposed to solve the problem. Here, we use the Lasso algorithm to get Equation (11) solved. In Section 4, more details about the parameter settings are given. Hence, we can get *L* sparse results and we synthesise them by regarding each $y_{k}$ as a column of the matrix Y and then $Y=[y_{0},y_{1},\ldots ,y_{L-1}]$ , which is called the results under the STSR method, which is

(12) Y=STSR(Φ,x).

If the signal received $s^{b}$ only contains $s^{r}$ ( or $s^{d}$), we only need to recover its motion parameters. Here, the $U^{l}$ that was built in Section 3.1 is used. Specifically, if only $s^{r}$ is contained, $U^{0}$ is used to recover the motion in time-frequency domain, which is

(13) $$a^{r}=\mathrm{STSR}\left(U^{0},s^{r}\right).$$

As for the case that only $s^{d}$ is contained, then the lag information $i=i_{0}$ is used to build the $U^{i_{0}}$ and the corresponding result is

(14) $$a^{d}=\mathrm{STSR}\left(U^{i_{0}},s^{d}\right).$$

The $U^{0}$ and $U^{i_{0}}$ are called true target dictionary and jamming dictionary, respectively. When the $s^{r}$ and $s^{d}$ exist simultaneously, we build a union dictionary $U_{\Sigma }=\left[U^{0}\;\;U^{i_{0}}\right]$ to process the back signal $s^{b}$:

(15) $$a^{\Sigma }=\mathrm{STSR}\left(U_{\Sigma },s^{b}\right).$$

$a^{\Sigma }$ can be obtained by applying Equation (11) and $a^{\Sigma }$ contains two parts, namely, the time-frequency estimation of two kinds of signals.

### 3.3. The Working Flow of the Anti-Velocity Deception Jamming Strategy

**Figure 5:**
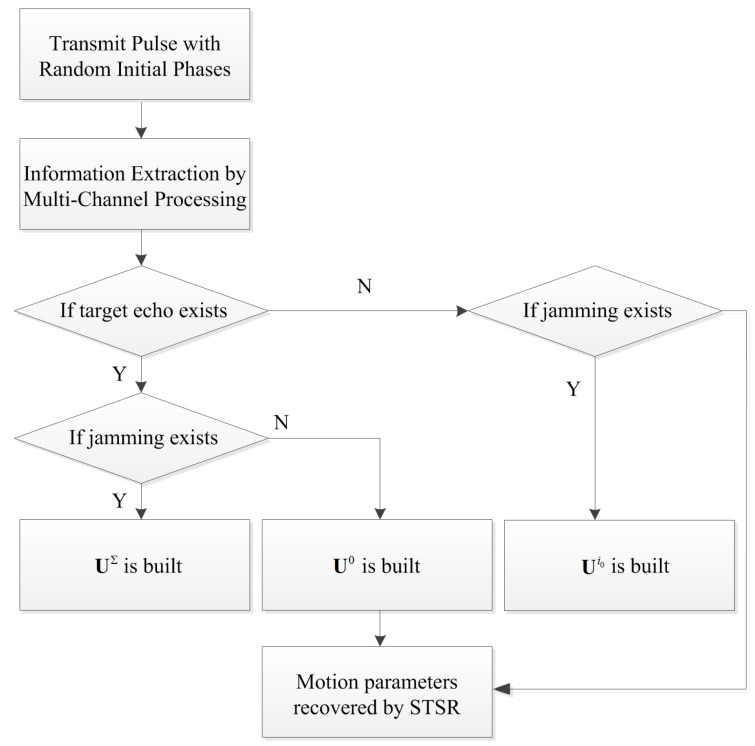
The working flow of the anti-velocity deception jamming strategy.

The proposed strategy mainly contains the following steps, that is, information extraction, dictionaries construction and motion parameters recovery. Figure 5 depicts the working flow of the anti-velocity deception jamming strategy.

As illustrated in Figure 5, the RPIP signal is transmitted firstly (denoted as $s^{t}$) and, if a moving target is detected, $s^{r}$ will be received by the radar. Simultaneously, the jammer may generate the jamming signal $s^{d}$. The signal that the radar received needs to be analyzed regarding its components firstly by using the multi-channel processing method. If $s^{r}$ and $s^{d}$ are contained, the true target dictionary and jamming dictionary will be built. When only $s^{r}$ or $s^{d}$ is contained, the corresponding dictionary will be built. Then, the STSR method is applied to recover the motion parameters in the time-frequency domain. Note that the working flow is valid for both TMFT and MMFT.

## 4. Numerical Simulations

In this paper, the performance and the superiority of the proposed strategy is tested by comparing with the traditional MTD and anti-velocity jamming method, namely, the CP-based method. Our simulations are performed in the MATLABR2016a environment (2016a, The MathWorks, Natick, MA, USA) using an Intel CPU 1.6 GHz processor with 8 GB of memory (Santa Clara, CA, USA). The convex optimization problems are solved by employing the SolveLasso function in cvx [31,32]. The parameter settings of the SolveLasso are: $algType{=}^{\prime }lasso^{\prime },maxIters=500,lamdaStop=0,resStop=0,solFreq=0,verbose=0,OptTol=epsi.$ The radar simulated in this paper works on X-band and transmits RPIP signal to detect the velocity target. The carrier frequency is 10 GHz, the PRI is 1kHz and the number of pulses in one CPI is 640.

**Figure 6:**
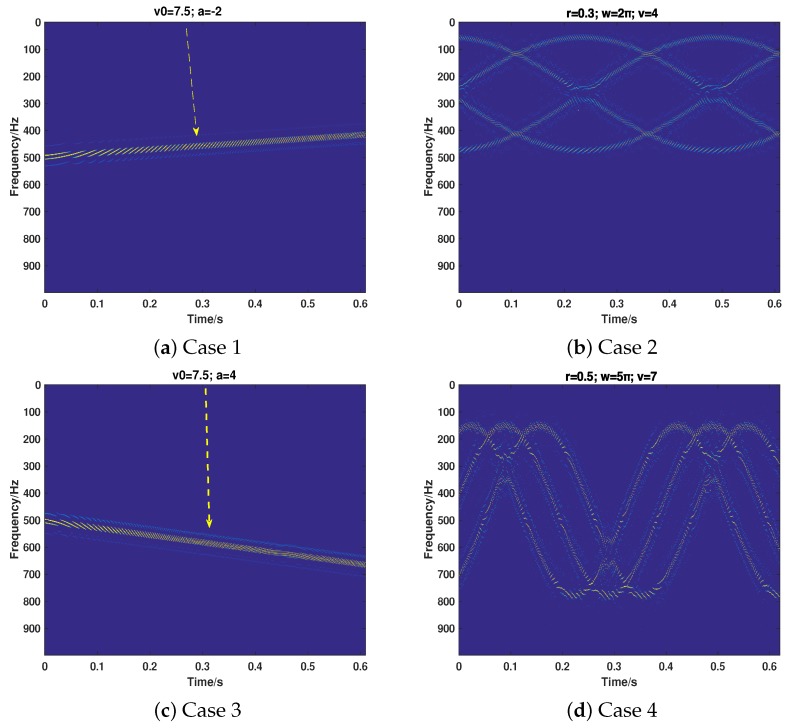
The corresponding results of Case 1–Case 4.

The simulation scenario is given here, which is demonstrated in Figure 6. Assume that there is a velocity target (denoted as $O^{r}$) and a jamming target ($O^{d}$) located in the radial direction of radar. Note that $O^{r}$ can be either a TMT or a MMT. Correspondingly, for a better jamming effect, $O^{d}$ will be a TMFT or a MMFT. Both the uniform linear motion (ULM) and uniform acceleration linear motion (UALM) are taken into consideration in the simulations. The instantaneous velocity, the initial velocity and the accelerated speed of $O^{r}$ ($O^{d}$) are denoted as $v^{r}$($v^{d}$), $v_{0}^{r}$($v_{0}^{d}$) and $a^{r}$($a^{d}$). Hence, for ULM cases, $R^{r}\left(t\right)=v^{r}t$ ($R^{d}\left(t\right)=v^{d}t$) and for UALM cases, $R^{r}\left(t\right)=v_{0}^{r}t+\frac{1}{2}a^{r}t^{2}$ ($R^{d}\left(t\right)=v_{0}^{d}t+\frac{1}{2}a^{d}t^{2}$). Because the rotation is one of the major micro motion forms [6,7], here we mainly consider the rotation as the micro motion form in our simulations without loss of generality. Assume that there are three (four) scattering centers rotating around the centroid of $O^{r}$ ($O^{d}$) and the corresponding radius and angular velocity of rotation are denoted as $r^{r}$ ($r^{d}$) and $\omega^{r}$ ($\omega^{d}$).

### 4.1. The Effectiveness of the Proposed Strategy

Here, the validity of our proposed method under different motion cases with regard to recognizing the target and separating the true motion target and false motion target is tested. It should be noted that the signal noise ratio (SNR) is 20 dB and the jamming signal ratio (JSR) is 7 dB. Firstly, we test its performance on the cases that only true motion targets or false motion targets are included. We consider that the motion of the true and false target can be either UALM or micro motion. The parameters are set as shown in Table 1. Case1 and Case 2 are the cases that only the true target echo is received and the motion can be either UALM (Case 1) or Micro Motion (Case 2). Case 3 and Case 4 take the case that only jamming signal is received. The corresponding results of Cases 1–4 are shown in Figure 6a–d. As shown in Figure 6, the proposed method can correctly identity the component of the received signal and recover the corresponding motion parameters.

**Figure 7:**
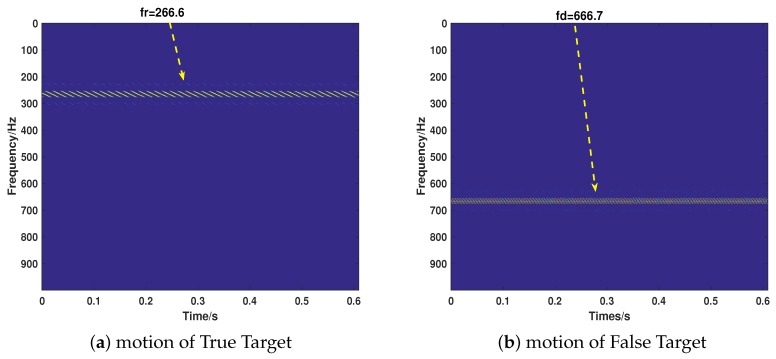
The recovery of motion of true (**a**) and false (**b**) target in Case 5.

**Figure 8:**
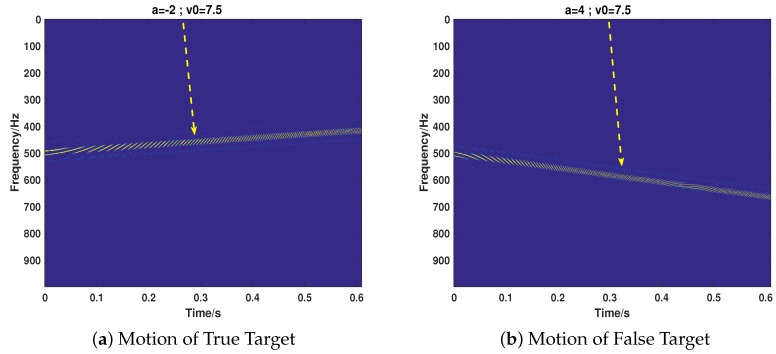
The recovery of motion of true (**a**) and false (**b**) target in Case 6.

**Figure 9:**
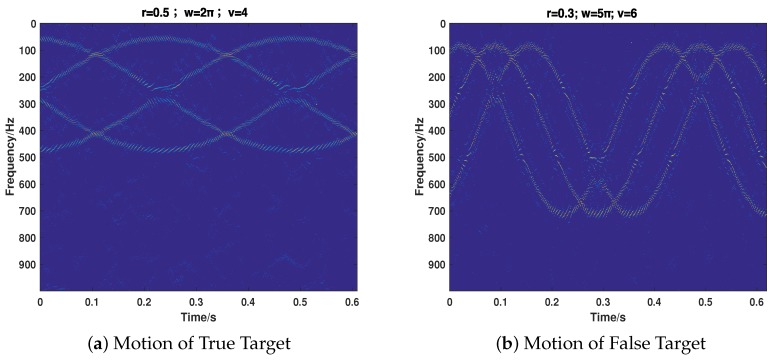
The recovery of motion of true (**a**) and false (**b**) target in Case 7.

**Figure 10:**
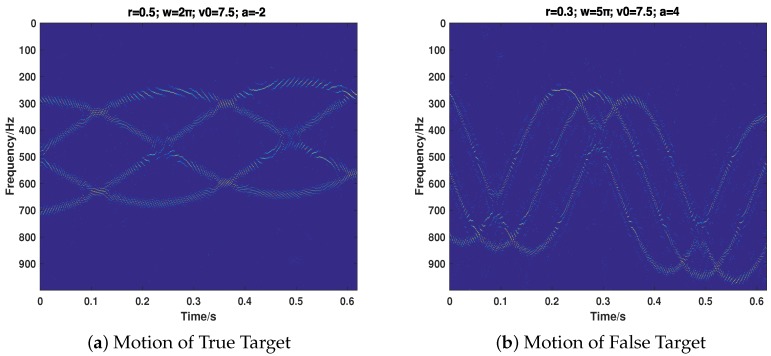
The recovery of motion of true (**a**) and false (**b**) target in Case 8.

Then, we consider the most common cases that the target echo and jamming signal exist simultaneously, putting emphasis on testing the proposed strategy’s performance of separating the two signals and recovering their motion parameters. We take the motion of the target into full consideration, which includes ULM (Case 5), UALM (Case 6), ULM with rotation (Case 7), and UAML with rotation (Case 8). The corresponding parameter settings are shown in Table 2. As can be seen from Figure 7, Figure 8, Figure 9 and Figure 10, the target echo and jamming signal can be separated accurately under all cases considered. The ULM motion will be recovered as a straight line in a time-frequency domain, as depicted in Figure 7. The velocity will be gained by applying the corresponding Doppler frequency into the Doppler formulation, namely, $f_{Dop}=2v_{Dop}/\lambda$ [6,7].

### 4.2. The Superiority of the Proposed Method

**Figure 11:**
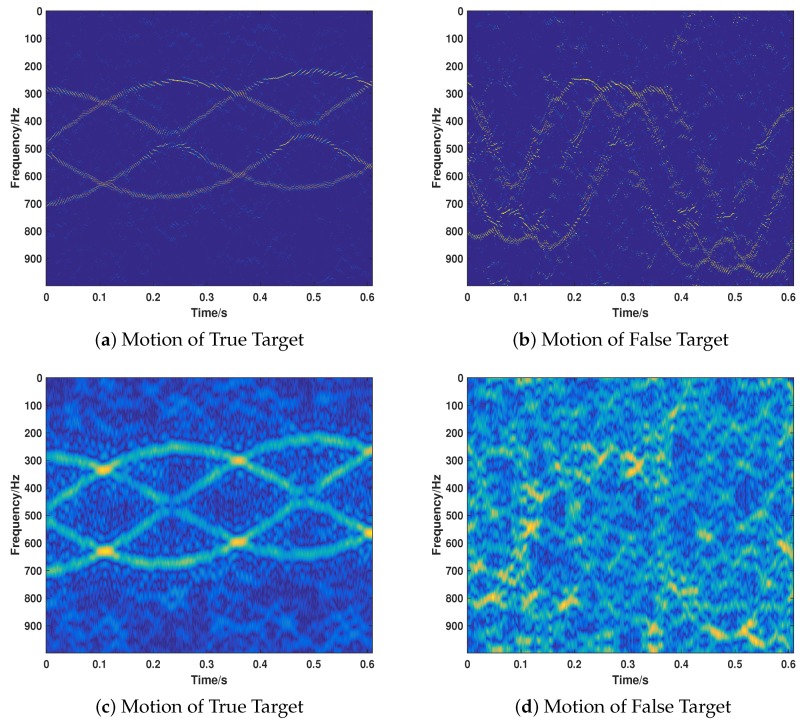
The motion of true and false target recovered by the proposed method (**a**,**b**) and the CP-based method (**c**,**d**) under JSR = −2 dB.

**Figure 12:**
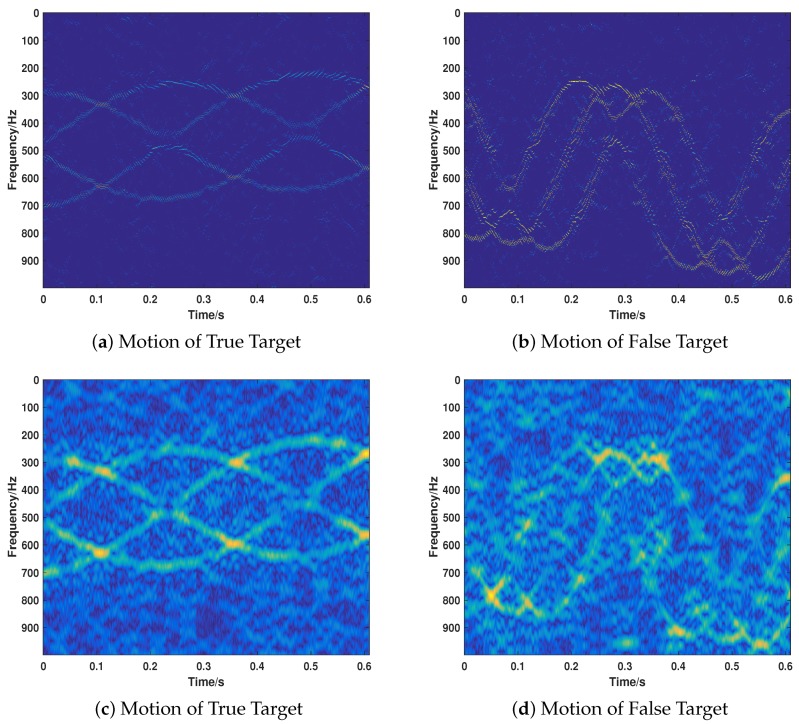
The motion of true and false target recovered by the proposed method (**a**,**b**) and the CP-based method (**c**,**d**) under JSR = 0 dB.

**Figure 13:**
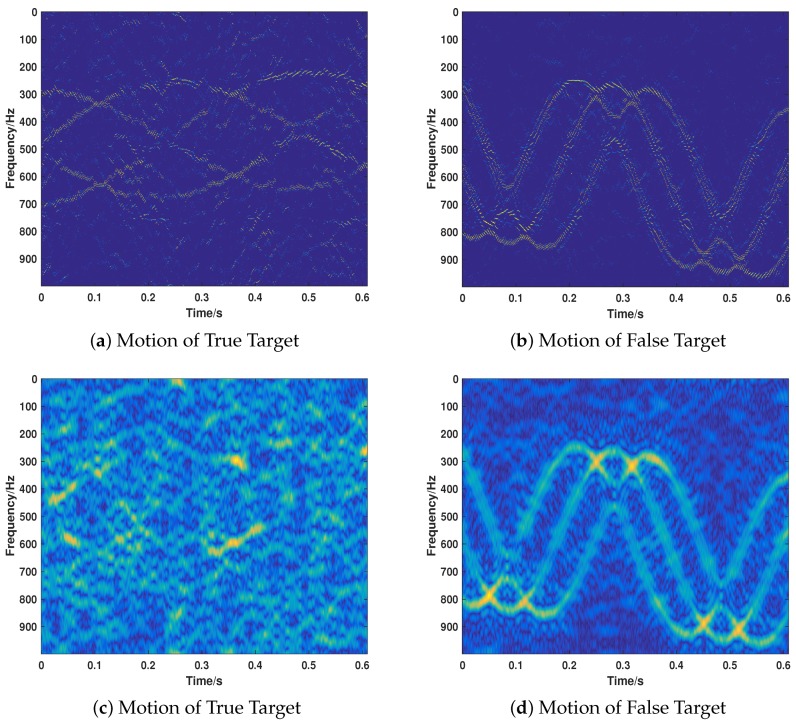
The motion of true and false target recovered by the proposed method (**a**,**b**) and the CP-based method (**c**,**d**) under JSR = 4 dB.

Due to the fact that most extant VDJ recognition methods are mainly based on CP theory, it is necessary to compare our proposed strategy with CP-based methods to show the superiority of our proposed method. However, considering the fact that most extant CP-based methods have no time-varying property, we modify the CP method slightly (called CP-based) according to the basic idea of STSR to get it sensitive to the time. As we have proven the correctness of the proposed strategy in different cases, we will only compare the performance of two methods under the same parameter settings of Case 8 for the sake of brevity. For a better comparison, we perform the simulations in different jamming intensity, that is, JSR=−2 dB, 0 dB, 4 dB. The corresponding results are illustrated in Figure 11, Figure 12 and Figure 13.

When the target is more intense than the jamming signal, the CP-based method can only recover the true signal (as shown in Figure 11c,d), while the proposed method can recover the two signals well with a much lower noise in the time-frequency domain. As shown in Figure 12, when the two kinds of signals are equivalent with regard to the intensity (JSR = 0 dB), the proposed method can recover the motion parameters accurately. However, the CP-based method cannot recover the motion of the jamming target. Moreover, the whole noise level is much higher than that of the proposed method, which is much more difficult for further researching. When the JSR = 4 dB, the CP-based method loses its effectiveness in recovering the motion parameters of the true target, as depicted in Figure 13. However, the proposed method keeps its validity under such jamming conditions. As can be seen from the results of Figure 11, Figure 12 and Figure 13, the CP-based method is more sensitive to the JSR conditions and it is easier to lose its effectiveness of the separation and recovery of the motion parameters for the true and false targets. By contrast, the proposed method is relatively robust in recognizing the true and false targets with more precise results and lower sidelobe noise floor for both true and false targets and a lower side-lobe noise base.

## 5. Conclusions

We study the problem of moving targets detection for PD radar in the presence of a translation/micro motion velocity false target. We propose an anti-velocity strategy based on sparse theory that can efficiently recognize the components of the received signal, recognize the false velocity target jamming if it exists, and accurately recover the motion parameters of true and false targets. Concretely, RPIP signal is transmitted by the radar and the difference between the target echo and jamming signal in phase domain is utilized. The strategy detects the components of the received signal by a multi-channel processing method based entropy and recovers the motion parameters in a time-frequency domain with the help of our previous work STSR. Compared with most extant measures, the key advantages of the proposed strategy are, firstly, not only the translational false target, but also the micro motion false target, which is a quite new trend in VDJ fields, are taken into account. Secondly, unlike current methods, assuming that the jamming and target echo exist simultaneously in the received signal, our approach considers more possibilities, namely only the target echo or jamming exists, or both of them are included. These possibilities are integrated into a unified framework and can be automatically detected and responded to by our strategy. Finally, we estimate the target parameters in the time-frequency domain based on sparse recovery theory and the recovery results are more accurate with a lower noise side-lobe base. Experiments show the effectiveness of our strategy and its superiority over the state of the art. It should be pointed out that, at present, our strategy is still in the simulation analysis and prototype testing stage. Future work includes real conditions verification and improvement of the prototype.

## Figures and Tables

**Table 1:** The corresponding parameter settings of Case 1–Case 4.

Case	True Motion	True Parameters	False Motion	False Parameters
Case 1	UALM	$v_{0}^{r}=7.5$ m/s; a${}^{r}=-2$ m/s${}^{2}$	–	–
Case 2	ULM with rotation	$r^{r}=0.5$ m; $w^{r}=2\pi$; $v^{r}=4$ m/s	–	–
Case 3	–	–	UALM	$v_{0}^{d}=7.5$ $m/s;a^{d}=4$ m/s${}^{2}$
Case 4	–	–	ULM with rotation	$r^{d}=0.5$ m; $w^{d}=5\pi ;$ $v^{d}=7$ m/s

**Table 2:** The corresponding parameter settings of Case 5–Case 8.

Case	True Motion	True Parameters	False Motion	False Parameters
Case 5	ULM	$v_{0}^{r}=4$ m/s	ULM	$v_{0}^{d}=7$ m/s
Case 6	UALM	$v_{0}^{r}=7.5$ m/s; $a^{r}=-2$ m/s${}^{2}$	UALM	$v_{0}^{d}=7.5$ m/s; $a^{d}=4$ m/s${}^{2}$
Case 7	ULM with rotation	$r^{r}=0.5\pi$ m; $w^{r}=2\pi ;$ $v^{r}=4$ m/s	ULM with rotation	$r^{d}=0.3$ m; $w^{d}=5\pi ;$ $v^{d}=6$ m/s
Case 8	UALM with rotation	$r^{r}=0.5$ m; $w^{r}=2\pi ;$ $v_{0}^{r}=7.5$ m/s; $a^{r}=-2$ m/$s^{2}\;\;$	UALM with rotation	$r^{d}=0.3$ m; $w^{d}=5\pi ;$ $v_{0}^{d}=7.5$ m/s; $a^{d}=4$ m/s${}^{2}\;\;$
